# THG113.31, a specific PGF2alpha receptor antagonist, induces human myometrial relaxation and BKCa channel activation

**DOI:** 10.1186/1477-7827-5-10

**Published:** 2007-03-16

**Authors:** Helen C Doheny, Michael J O'Reilly, Donal J Sexton, John J Morrison

**Affiliations:** 1 Department of Obstetrics & Gynaecology, National University of Ireland Galway, Clinical Science Institute, University College Hospital Galway, Newcastle Road, Galway, Ireland

## Abstract

**Background:**

PGF2alpha exerts a significant contractile effect on myometrium and is central to human labour. THG113.31, a specific non-competitive PGF2alpha receptor (FP) antagonist, exerts an inhibitory effect on myometrial contractility. The BKCa channel is ubiquitously encountered in human uterine tissue and plays a significant role in modulating myometrial cell membrane potential and excitability. The objective of this study was to investigate potential BKCa channel involvement in the response of human myometrium to THG113.31.

**Methods:**

Single and whole-cell electrophysiological BKCa channel recordings from freshly dispersed myocytes, were investigated in the presence and absence of THG113.31. Functional studies investigated the effects of THG113.31 on isolated spontaneous myometrial contractions, in the presence and absence of the BKCa channel blocker, iberiotoxin.

**Results:**

Single channel recordings identified the BKCa channel as a target of THG113.31. THG113.31 significantly increased the open state probability of these channels [control 0.023+/-0.006; 10 microM THG113.31 0.087+/-0.012 (*P *= 0.009); and 50 microM THG113.31 0.1356+/-0.018 (*P *= 0.001)]. In addition, THG113.31 increased whole-cell BKCa currents over a range of membrane potentials, and this effect was reversed by 100 nanoM IbTX. Isometric tension studies demonstrated that THG113.31 exerted a significant concentration-dependent relaxant effect on human myometrial tissue and pre-incubation of strips with IbTX abolished this effect on spontaneously occurring contractions.

**Conclusion:**

These data suggests that activation of the BKCa channel may contribute, at least partially, to the uterorelaxant effect of THG113.31.

## Background

The mechanisms underlying the onset and maintenance of human parturition are poorly understood [1,2]. During pregnancy and labour the uterus undergoes dramatic changes in its contractile activity when it is transformed from a relatively quiescent state during pregnancy to a state of maximal contractile activity at labour onset. The exact regulatory mechanisms for this process are unclear [3,4], but what is well established, is that abnormalities of this process have major clinical implications. Preterm labour is the single largest cause of perinatal mortality in developed countries and is a major contributor to neonatal morbidity and childhood developmental problems [1,5,6]. In addition, these problems constitute an immense cost to healthcare resources.

PGF_2α _plays a pivotal role in parturition [7-9]. It is the most effective smooth muscle contractile prostaglandin [7], which exerts a significant stimulatory effect on the myometrium [10], and is a potent compound for inducing labour [8]. A significant rise in PGF_2α _levels occurs in the amniotic fluid during early labour [11]. A rise in PGF_2α _levels results in stimulation of myometrial contractions in various species [10,12]. Inhibition of prostaglandin formation results in reduced myometrial contractions and prolonged labour [13] and disruption of the PGF_2α _receptor gene leads to failure of animals to go into labour [14]. THG113 has recently been reported as a selective, non-competitive, reversible, and potent FP receptor antagonist [8,9]. A version of the FP receptor antagonist, THG113.31 has recently been reported to significantly delay preterm labour in an endotoxin-induced preterm labour mouse model [8], providing the basis for future investigations for its use in tocolysis. In vitro functional studies, from our laboratory, support this tocolytic effect by demonstrating that THG113.31 exerted a potent concentration dependent relaxant effect on both spontaneous and oxytocin induced contractions, in human myometrial tissue [15,16].

The nature by which THG113.31 inhibits contractions in the uterus has yet to be elucidated. A primary determinant of smooth muscle tone and contractility is the resting membrane potential, which, in turn, is influenced heavily by K^+ ^channel activity [17,18]. The large conductance calcium-activated potassium channel (BK_Ca_) is the predominant K^+ ^channel type in both non-pregnant and pregnant human myometrium [19-23] and plays an important role in suppressing myometrial activity during gestation [22]. This channel is considered to be relatively easy to identify and study, using electrophysiological single channel and whole cell patch clamp methodologies due to its large single channel conductance (> 200 pS in symmetrical K^+ ^gradients) and its pharmacological sensitivity to the extracellular blockers tetraethylammonium, charybdotoxin and iberiotoxin (IbTX).

We sought to investigate whether the BK_Ca _channel was a target site for THG113.31 activity. Using single channel electrophysiological recordings we determined the effects of THG113.31 on the open state probability of BK_Ca_channels in human uterine myocytes, and evaluated if the relaxant effect of THG113.31 on spontaneous myometrial contractions was altered by BK_Ca _blockade, in functional studies, using IbTX.

## Materials and methods

### Tissue collection

Biopsies of human myometrium were obtained from 26 women undergoing elective cesarean section in the third trimester of pregnancy, in the Department of Obstetrics and Gynecology, University College Hospital, Galway, Ireland. The reasons for caesarean section included breech presentation, previous cesarean section and medical conditions, such as maternal spina bifida, maternal pelvic disorder and placenta praevia. All cesarean sections were performed prior to the onset of labour under regional anesthesia. The maternal demographic details were as follows: mean age 34 years (range 22–42); median period of gestation 39 weeks (range 37–40); and median parity 1 (range 0–5).

Biopsies were excised from the midline portion of the upper lip of the incision in the lower uterine segment. Women who had received exogenous prostaglandins, oxytocin or corticosteroids were excluded from the study. Recruitment was by written informed consent. Ethics committee approval for the study was obtained from the Research Ethics Committee, University College Hospital, Galway. For electrophysiological studies tissue samples were immediately placed in sterile Ham's F-12 medium (Sigma-Aldrich, Dublin, Ireland), supplemented with 100 Uml^-1 ^penicillin and 100μg ml^-1 ^streptomycin (Sigma-Aldrich, Dublin, Ireland). Tissue samples for isometric recordings were placed in fresh Krebs-Henseleit physiologic saline solution (PSS) of the following composition (mM): 4.7 KCl, 118 NaCl; 1.2 MgSO_4_, 1.2 CaCl_2_, 1.2 KH_2_PO_4_, 25 NaHCO_3_, and 11 glucose (Sigma-Aldrich, Dublin, Ireland). Tissue was stored at 4°C and used within 6 hours of collection.

### Tissue dispersion/primary human myometrial cells

The preparation of single myometrial cells for electrophysiological recordings was performed using methodology previously described [24]. Freshly isolated myometrial cells were obtained by enzymatic digestion of finely minced myometrium with 2 mg ml^-1 ^collagenase (type IA, 300–400 U mg^-1^) (Sigma-Aldrich, Dublin, Ireland) in Hanks buffered salt solution. The incubation with enzyme was performed @ 37°C for 2 hrs followed by centrifugation (1000 revs min^-1^) in 50% Percoll (Sigma-Aldrich, Dublin, Ireland) for 10 minutes. The cell layer was removed, washed, and spun in physiological solution to remove excess red blood cells. The cell suspension was then triturated and filtered through an 80 μm nylon mesh filter. Single cells were placed in a recording chamber (Warner Instrument Corporation, Hamden, CT 06514, USA) and electrophysiological experiments begun immediately. Morphologically, freshly dissociated uterine myocytes were characterized by a long, slender fusiform shape. All myocytes used for this work were relaxed and adhered to the bottom of the recording chamber with no additional substrate.

### Electrophysiological recordings of the BK_Ca _channel

Single channel recordings using the cell-attached configuration of the patch clamp technique were performed. Several drops of cell suspension were placed in the recording chamber containing a solution of the following composition (mM): 140 KCl, 10 MgCl_2_, 0.1 CaCl_2_, 10 HEPES, and 30 glucose (pH 7.4, 22–25°C). Single potassium channel currents were measured in cell-attached patches by filling the patch pipette (2–5 MΩ) with Ringer solution composed of (mM); 140 NaCl, 5 KCl, 1 MgCl_2_, 2 CaCl_2_, and 10 HEPES, and making a gigaohm seal on a single myocyte. Voltage across the patch was controlled by setting the cellular membrane potential to 0 mV using a high- [K^+^] extracellular solution. Average channel activity in patches was measured as mean open probability (NP_o_). The effects of THG113.31 (10 and 50 μM) on channel activity were measured by recording ~10 seconds of continuous recording at +40 mV, before and 15 minutes after drug treatment. Currents were filtered at 1 kHz and digitised at 10 kHz. In all experiments voltage-clamp and voltage-pulse generation were controlled with an Axopatch 200-A patch-clamp amplifier (Axon Instruments, Union City, California, USA), and data was acquired and analysed with pClamp 8. For statistical purposes, and to ensure accuracy, NP_o _was calculated for single BK_Ca _channels at a depolarised potential where channel openings can be clearly differentiated from other channel species. BK_Ca _channels are easily visualised at + 40 mV as there is virtually no "contamination" with other channel species [25]. In addition, at such depolarised voltages, there is no other ion channel expressed in these cells with an amplitude of 7–9 pA, making statistical analysis of BK_Ca _channel activity more accurate [25]. Moreover, recording single channel activity at more depolarised potentials helps compensate the artificial depression of activity otherwise encountered from recording channel activity at room temperature (22–25°C) [25].

In a separate set of experiments the direct effect of PGF_2α _on BK_Ca _channel activity was investigated. Cells were pre-incubated with 20 μM NS1619, the specific channel opener, for 15 minutes before cell-attached recordings were taken and the effect of PGF_2α_, in the presence of NS1619 was determined (n = 4). Furthermore, in additional experiments, myometrial cells were pre-incubated with 1 μM PGF_2α_, cell-attached recordings were taken and the effect of 10 μM THG113.31 on channel activity was again measured (n = 3).

For perforated patch experiments, the recording chamber contained a bath solution of the following composition (mM): 140 NaCl, 5 KCl, 1 CaCl_2_, 2 MgCl_2_, 10 HEPES, and 30 glucose (pH 7.4; 22–25°C). To measure potassium currents, the tip of the patch pipette (1–5 mM) was filled with a solution containing (mM): 60 K_2_SO_4_, 30 KCl, 5 MgCl_2_, 5 CaCl_2_, 5 mm HEPES, and 40 mm MgSO_4 _(pH 7.4). Because divalent ions do not pass through pores in the perforated membrane, the high Ca^2+ ^in the pipette solution does not enter the cell, thus obviating the need for artificial Ca^2+ ^buffers. In contrast, the K^+ ^channels were always exposed to physiological levels of Ca^2+ ^(bath solution) in these whole-cell recording experiments and not the 5 mmol of the pipette solution. The remainder of the pipette was back-filled with the same solution to which 6 mg/ml amphotericin B (diluted by sonication from a 50 mg/ml stock in dimethylsulfoxide) was added. Generation of voltage clamp protocols and acquisition of data were carried out using pClamp software (version 8). Voltage-activated currents were filtered at 1 kHz and digitized at 10 KHz. Leakage currents were algorithmically subtracted using short duration, small amplitude negative prepulses.

### Isometric tension recordings

Longitudinal myometrial strips (measuring approximately 2 × 2 × 10 mm) were dissected free from uterine decidua and serosa and mounted isometrically in an organ tissue bath under 2 g tension for recordings, as previously described [24]. The tissue baths contained 20 ml of Krebs-Henseleit PSS maintained at 37°C, pH 7.4, and were gassed continuously with a mixture of 95% oxygen/5% carbon dioxide. During a period of equilibration of one hour, the Krebs-Henseleit PSS in the tissue baths was changed every 15 minutes. The experiments evaluating effects on spontaneous myometrial contractility were designed into 4 subgroups as follows: *Group 1*. spontaneous alone; *Group 2*. spontaneous + THG113.31; *Group 3*. spontaneous + IbTX; *Group 4*. spontaneous + IbTX + THG113.31. After 30 minutes, strips from Groups 3, and 4 were exposed to IbTX at a bath concentration of 100 nM for a further 30 minutes, while Group 1 and 2 strips were exposed to Krebs-Henseleit PSS only. Contractile activity was measured for a 20- minute period at which time bath addition of THG113.31 to Group 2 and 4 strips took place. Highly purified THG113.31 was added to the tissue bath in a cumulative manner at bath concentrations 1 nM, 10 nM, 100 nM, 1 μM and 10 μM at 20 minute intervals, and the resultant contractile activity was measured for each period and expressed as a percentage of the integral obtained in the 20 minute period prior to any THG113.31 addition (i.e. percentage of contractility). Measurement of contractile activity was performed by calculation of the integral of the selected area with the PowerLab (AD Instruments, Hastings, UK) hardware unit and Chart v3.6 software (AD Instruments, Hastings, UK).

### Drugs and solutions

THG113.31 was obtained as a gift from Dr Krishna Peri, Theratechnologies, Quebec, Canada. A stock solution (1 mM) was made in deionised water. Serial dilutions were prepared in deionised water and stored at room temperature. A stock solution of IbTX (Sigma-Aldrich, Dublin, Ireland) (1 × 10^-5 ^M) was made in saline. Fresh Krebs-Henseleit PSS was prepared daily. All other chemicals were obtained from Sigma-Aldrich, Dublin, Ireland.

### Data and statistical analysis

For single channel activity data NP_o _values are expressed as mean ± standard error of the mean. Comparisons between groups were made by one-way analysis of variance (ANOVA), followed by a post-hoc Tukey HSD test to determine significant differences among data groups. Currents were filtered at 2 kHz and digitized at 10 kHz. Average channel activity in patches with multiple BK_Ca _channels determined by:

*NP*_*O *_= *Σ*^n^_j = 1 _t_j _J/T

where P_o _is the single-channel open-state probability, T is the duration of the recording, tj is the duration of j = 1,2, ..... n channel openings, J is the number of channels open for duration tj, and N is the maximal number of simultaneous channel openings observed when P_o _was high.

For isometric recordings, using the calculated integrals of contractile activity at each bath concentration dose response curves were analysed by fitting the sigmoidal dose-response equation:

Y = Y_max _x D^n^_H_/EC_50 _+ D^n^_H_

Where Y is the response (percentage contractility), Y_max _is the maximal relaxation achieved, D is the dose of agonist (THG113.31), ^n^_H _is the slope function, and EC_50 _is the agonist dose giving the half maximal response. Curve fitting was performed with the software package Prism (Graphpad Software, San Diego, California, USA). Multiple comparisons of measured integrals of contractility were performed using two-way ANOVA, followed by post-hoc Tukey test. The statistical packages SPSS Version 11.0 (Chicago, Illinois, USA) and SigmaStat Version 2.0 (SPSS Inc., Chicago, USA) were used for statistical calculations. A *P *value < 0.05 was accepted as statistically significant.

## Results

### BK_Ca _channel identification

**Figure 1:**
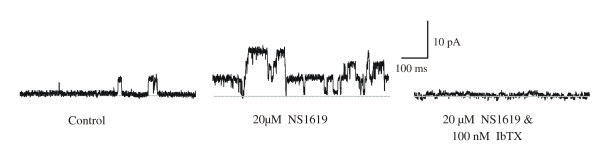
NS1619, a putative activator of BK_Ca _channels, and IbTX, a specific BK_Ca _channel blocker, serve to identify the BK_Ca _channel in human uterine smooth muscle myocytes. Representative recordings from the same membrane patch (+40 mV) in the cell-attached configuration before and after addition of 20 μM NS1619 and subsequent addition of 100 nM IbTX. IbTX reversed NS1619-stimulated channel activity.

Single-channel recordings from freshly dispersed myocytes, using the cell-attached configuration of the patch clamp technique, revealed membrane electrical activity to be dominated by a prominent, large conductance (152 ± 19.30 pS (n = 6) physiological gradients of potassium; range 115–206 pS) channel carrying outward potassium currents. Channel activity recorded, from a cell attached patch, with an amplitude range of 6–10 pA at +40 mV, under control conditions revealed minimal gating events (NP_o _= 0.034 ± 0.006; n = 3). In contrast, exposure to NS1619, a specific BK_Ca _channel opener, elicited potent channel activation at applied concentration of 20 μM, (0.103 ± 0.0196 (n = 3); (Figure 1)). Subsequent addition of IbTX (100 nM), a specific extracellular BK_Ca _channel blocker, reversed NS1619-stimulated channel activity. These properties, the conductance value, NS1619- and IbTX- sensitivity are in accordance with the characteristics of the BK_Ca _channels in smooth muscle myocytes described in literature [26,27]. Therefore, we identified this protein as the high-conductance, NS1619-and IbTX- sensitive BK_Ca _channel, which is the predominant K^+ ^channel species in human myometrial smooth muscle [19-23].

### BK_Ca _channel activity

**Figure 2:**
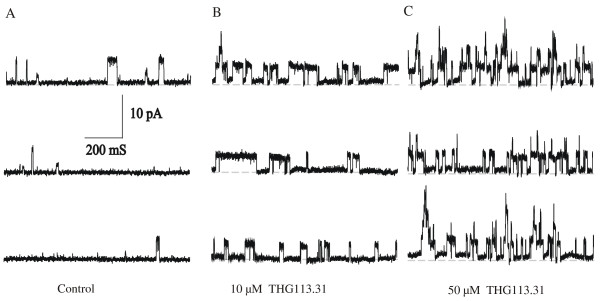
THG113.31 enhances BK_Ca _channel activity in human myometrial smooth muscle cells. **A, B & C **Representative continuous recordings were recorded from the same cell attached patch (+40 mV) before (control) and 15 min after exposure to 10 & 50 μM THG113.31, respectively. Channel openings are upward deflections from the baseline (closed state) (dashed line).

**Figure 3:**
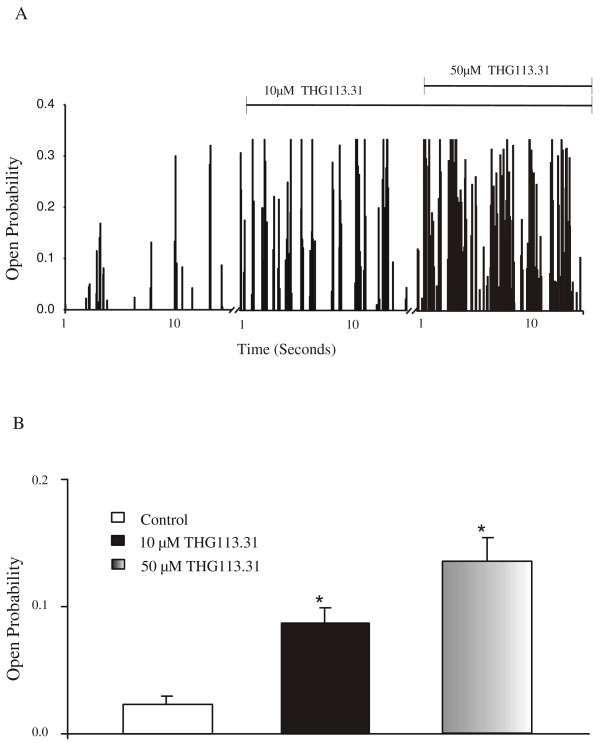
THG113.31 opens BK_Ca _channel activity in human myometrial smooth muscle cells in a concentration-dependent manner. **A **Activity plot of BK_Ca _channel open probability in a single cell-attached patch before and 15 min after exposure to cumulative doses of 10 and 50 μM THG113.31. Vertical bars are channel activity during a 100-ms test pulse to +40 mV. Recording time under each condition was 10 s, as indicated on the time axis. Breaks in the time axis represent drug incubation periods when channel activity was not recorded. Period of drug exposure is indicated by horizontal lines above the histogram. **B **Each bar represents the average channel open probability obtained from cell attached patches (+40 mV) before and 15 min after additions of 10 and 50 μM THG113.31, respectively. *Significant increase in channel activity compared to control (n = 6; *P *< 0.005)

A representative recording, demonstrating minimum BK_Ca _channel activity under control conditions (NP_o _= 0.022), is shown in Figure 2A. Cumulative additions of THG113.31 resulted in a potent activation of BK_Ca _channel activity in a concentration-dependent fashion, as illustrated (Figure 2B &2C). BK_Ca _channel activity for this recording was increased following the addition of 10 (NP_o _= 0.102) and 50 (NP_o _= 0.191) μM THG113.31, respectively (Figure 3A). The average NP_o _(Figure 3B) before and after addition of THG113.31 was as follows: control 0.023 ± 0.007 (*n *= 6); 10 μM THG113.31 0.087 ± 0.012 (*n *= 6; *P *= 0.005) and 50 μM THG113.31 0.136 ± 0.018 (*n *= 6; *P *= 0.001). THG113 .31 stimulated the BK_Ca _channel with a maximal activation effect observed at the highest concentration of 50 μM THG113.31. Open state channel activity was stimulated on average 4 and 6-fold by 10 and 50 μM THG113.31 respectively. In general, there was a 5–10 minute latency period prior to observation of THG113.31-stimulated channel activity, and this effect appeared to be maximal within 10–15 minutes. In all patches this activity persisted until either seal integrity was lost, or the experiment was terminated.

**Figure 4:**
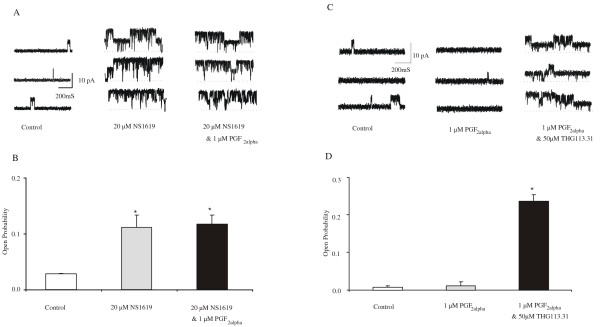
PGF_2α _has no effect on BK_Ca _channel activity. **A **Representative recordings from the same membrane patch (+40mV) in the cell-attached configuration after addition of 20 μM NS1619 and subsequent addition of 1 μM PGF_2α_. PGF_2α _did not reverse NS1619-stimulated channel activity. **B **Each bar represents the average channel open probability obtained from cell attached patches (n = 4; +40 mV) before and 15 min after each addition (n = 4). *Significant increase in channel activity compared to control. **C & D **THG113.31 activates the BK_Ca _channel independently of the FP receptor. Representative recordings from the same membrane patch (+40mV) in the cell-attached configuration before and after addition of 1 μM PGF_2α _and subsequent addition of 10 μM THG113.31. Each bar represents the average channel open probability obtained from cell attached patches (n = 3; +40 mV) before and 15 min after each addition (n = 3). *Significant increase in channel activity compared to control.

A further set of experiments on cell-attached patches indicated that the stimulatory effect observed with THG113.31 was not mediated via the PGF_2α _receptor (Figure 4). PGF_2α _failed to have any effect on BK_Ca _channel activity after pre-icubation of the cells with NS1619 as seen in Figure 4A. The average NP_o _before and after addition of NS1619 and further application of PGF_2α _was as follows: control 0.028 ± 0.001 (n = 4), 20 μM NS1619 0.111 ± 0.022 (n = 4; *P = 0.016*), 1 μM PGF_2α _0.142 ± 0.03 (n = 4; *P = 0.04*). The average effects are summarized in Figure 4B. Furthermore, pre-incubation with PGF_2α _had no effect on the stimulatory effect observed with THG113.31 as illustrated in Figure 4C and 4D. The typical response to THG113.31 in the presence of PGF_2α_, is illustrated by the traces in Figure 4C. The average effects are summarized in Figure 4D. BK_Ca _channel activity remained unchanged after 15 minutes exposure to PGF_2α_, when compared to control channel activity. The average NP_o _before and after addition of PGF_2α _and further application of THG113.31 was as follows: control 0.01 ± 0.001 (*n *= 3), 1 μM PGF_2α _0.015 ± 0.005 (*n *= 3; *P *= 0.99) and 10 μM THG113.31 0.135 ± 0.007 (*n *= 3; *P *= 0.0002).

**Figure 5:**
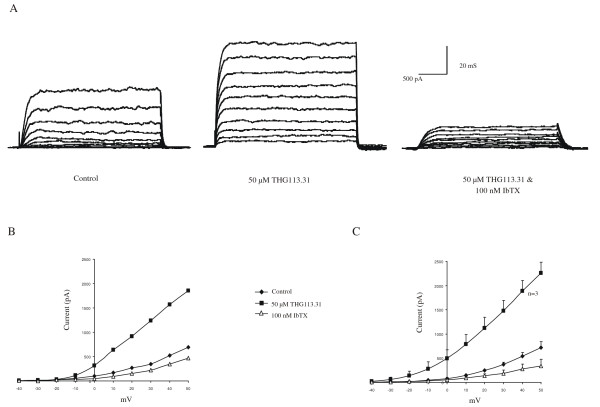
THG113.31 increases whole-cell currents in human uterine smooth muscle myocytes. **A **Perforated patch recordings (n = 3) from the same myocyte before and 15 min after exposure to 50 μM THG113.31, and then 5–10 min after cumulative addition of 100 nM IbTX. Tracings were taken from a range of potentials (-40 to +50 mV), with a holding potential of -60 mV. **B **The complete current (pA)-voltage (mV) relationship for steady-state outward current from the same cell as in A. Treatment conditions are the same as described for control, 50 μM THG113.31, and THG113.31 and 100 nM IbTX. **C **Average current-voltage relationship for uterine myocytes before and after 50 μM THG113.31, and then cumulative addition of 100 nM IbTX. Each point represents the mean number of cells ± SEM.

Whole-cell studies from single human uterine myocytes were performed to further characterize the effects of BRL37344 on ionic currents. As illustrated in Fig. 1, uterine myocytes exhibited prominent outward currents. Application of 50 μM THG113.31 increased these steady-state outward currents at all positive voltages (Figure 5A &5B). A complete current-voltage relationship illustrating the stimulatory effect of THG113.31 is presented in Figure 5B. This effect of THG113.31 was observed at all voltages where outward current was elicited. The effect of THG113.31 was reversed by 100 nm IbTX. A summary of the stimulatory effect of 50 μM THG113.31 followed by application of IbTX is presented in Figure 5C (n = 3). These whole-cell studies clearly demonstrate that THG113.31 stimulates outward current in human uterine smooth muscle cells, and blockage of these outward currents with IbTX strongly suggests the involvement of the BK_Ca _channels.

### In vitro contractility

**Figure 6:**
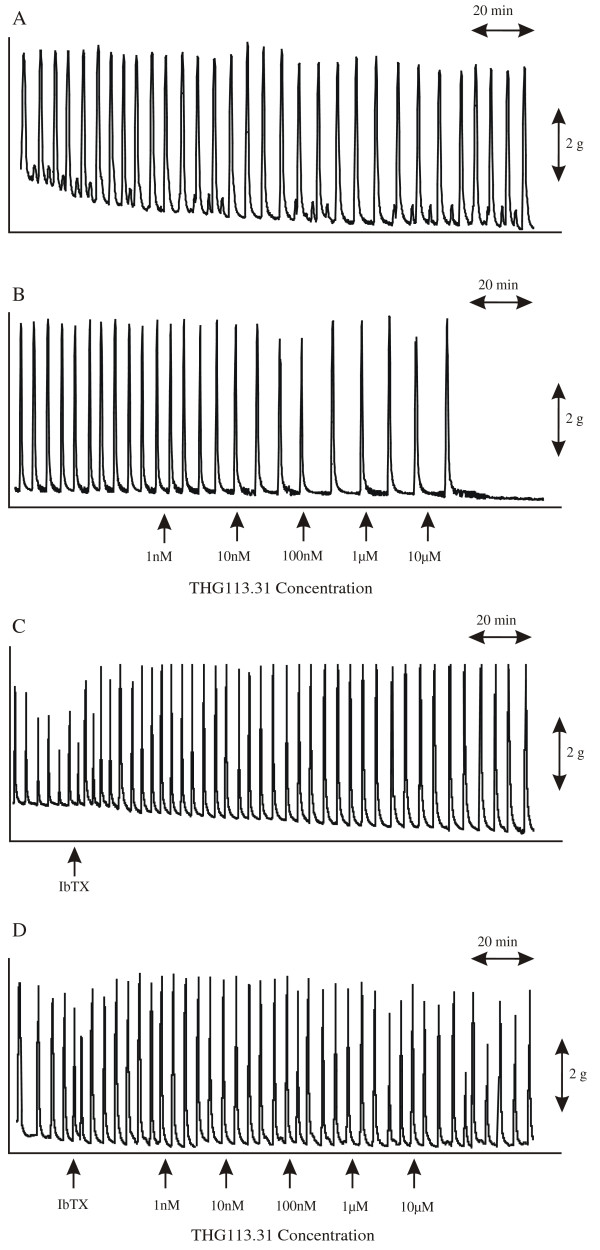
Effects of THG113.31 on spontaneous myometrial contractions. **A **Representative recordings of spontaneous contractions of pregnant human myometrium under control conditions and **(B) **the effects of cumulative additions of THG113.31 (1 nM, 10 nM, 100 nM, 1 μM and 10 μM) at 20 min intervals are shown. **(C & D) **The uterorelaxant effect was significantly attenuated by pre-incubation with the BK_Ca _channel antagonist, IbTX

THG113.31 exerted a cumulative inhibitory effect on spontaneous contractions in isolated pregnant human myometrium (*n *= 6). Figure 6A demonstrates a representative recording of spontaneous myometrial contractility and figure 6B the relaxant effects of cumulative additions of THG113.31 (1 nM, 10 nM, 100 nM, 1 μM and 10 μM). Analysis of variance revealed a significant difference in integrals of contractility measured across the groups of experiments which on post hoc testing revealed a statistically significant relaxant effect of THG113.31 at bath concentrations of 10 nM (n = 6; P < 0.001), 100 nM (n = 6; P < 0.001) and 1 μM (n = 6; P < 0.001) and 10 μM THG113.31 (n = 6; P < 0.001) when compared to respective control values measured. Pre-incubation of strips with IbTX, did not significantly alter the integrals of contractile activity measured (Group 1 versus Group 3; n = 6; *P *= 0.10), although an increasing trend in frequency was observed as seen in Figure 6C. Bath exposure of the strips to IbTX, prior to addition of THG113.31, abolished the inhibitory effect of THG113.31. Figure 6D represents a recording demonstrating the effects of pre-incubation with IbTX, on the uterorelaxant effects of THG113.31. Comparison of the inhibitory effects of THG113.31 on spontaneous contractions of myometrial strips, in the presence or absence of IbTX, revealed a significant difference across the groups (Group 2 versus Group 4; n = 6; *P <*0.05). Post hoc analysis demonstrated that IbTX (100 nM) significantly attenuated the uterorelaxant effect of THG113.31 at all test concentrations, resulting in no significant difference when compared with control values (Group 3 (n = 5) versus Group 4 (n = 6); *P *> 0.05) at all THG113.31 bath concentrations.

**Figure 7:**
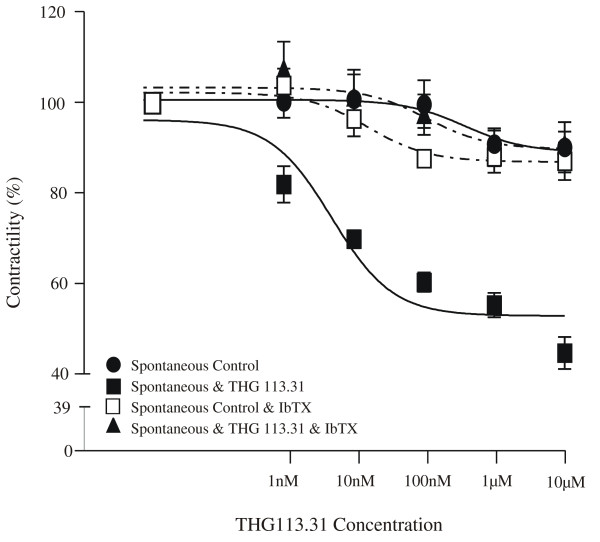
Dose response curves for THG113.31 and spontaneous myometrial contractions. Graphical representation of the effects of cumulatively increasing tissue bath concentrations of THG113.31 (1 nM, 10 nM, 100 nM, 1 μM and 10 μM) at 20 min intervals on spontaneous (shaded circle) contracting pregnant myometrium in the presence (shaded triangle) and absence (shaded square) of IbTX. A control trace showing uninterrupted spontaneous contractions in pregnant myometrium in the presence (open square) and absence (shaded circle) of IbTX, are shown for comparison. The symbols used represent the mean values (n = 6) within each group. Vertical error bars represent the standard error of the mean.

In Figures 7, the mean effect of cumulative additions of THG113.31 on spontaneous (n = 6) contractions of myometrial strips, in the presence and absence of IbTX, are shown in graphical form alongside the integrals observed in control strips.

## Discussion

The results of several studies have suggested that PGF_2α _receptors are present in the uterus and play a pivotal role during human labour. The findings from the present study demonstrated that THG113.31 induced a rapid, concentration dependent relaxation on spontaneous human uterine contractions. These data provide evidence that activation of the BK_Ca _channels significantly contributed to the relaxant effect of THG113.31 in the human myometrium. Myometrial smooth muscle cells are richly endowed with BK_Ca _channels [19,28,29] and these channels are important regulators of smooth muscle contractility [19]. Using the cell-attached configuration of the patch clamp technique, THG113.31 significantly increased the open state probability of these channels, in a concentration-dependent manner. Using the whole-cell configuration, similar findings were confirmed. These results concur with the data obtained from the isometric tension recordings. In the functional studies, the uterorelaxant effect mediated by THG113.31 was evident on spontaneously occurring contractions, in human myometrium, and was significantly attenuated by BK_Ca _channel blockade. Hence following pre-incubation with IbTX, the contractility measured after THG113.31 exposure was statistically different to that of spontaneous control strips i.e. strips allowed to contract spontaneously in the presence or absence of IbTX. It is therefore evident that THG113.31 activates BK_Ca _channel activity. However, our findings demonstrate no functional coupling of PGF_2α _receptors to BK_Ca _channel activity from freshly isolated human myocytes, using cell-attached single channel recordings, despite THG113.31 significantly increasing the open state probability of these channels in a concentration-dependent manner. One explanation is that the effect of THG113.31 on these channels has little to do with its activity at the FP receptor. PGF_2α _was demonstrated to have no direct effect on the activity of these BK_Ca _channels. The most plausible conclusion is that the BK_Ca _channel effect of THG113.31 exists independently of its putative PGF_2α _blockade role.

These findings, therefore, indicate that PGF_2α _receptor antagonism induces uterorelaxation, at least partially, via activation of BK_Ca_channel activity. The possibility of other signalling pathways and/or other mechanisms of action, however, do also exist. The other effector mechanisms or signalling pathways involved in this process, however, were not addressed during this study. This remains an exciting study requiring further evaluation.

Spontaneous contractions indigenous to the smooth muscle, may occur at different frequencies in myometrial smooth muscle strips. For this reason control experiments were run simultaneously for all strips. It is our view that contractions indigenous to the myometrium, once properly controlled for, provide a good model of in vitro contractility in investigating a utero-relaxant compound and its potential antagonist. The other option is to use a model of agonist- (eg. oxytocin, endothelin) induced contractions, which are commonly used [30] but for these experiments, where IbTX was also added, it was preferable to use spontaneous contractions only.

## Conclusion

In conclusion, this is the first report which demonstrates that THG113-mediated relaxation of human pregnant myometrium appears to involve, at least in part the opening of uterine BK_Ca _channels. However, this uterorelaxant effect of THG113.31 does not appear to be functionally linked to PGF_2α _or its receptor, FP. Finally, these findings support the possibility that THG113.31 may confer therapeutic benefit in preterm labour management, by a mechanism that appears to be mediated by BK_Ca _channel activity.

## Authors' contributions

HCD designed and performed the electrophysiological experiments, analysed the data and wrote the manuscript. MWOR and DJS performed the tension study experiments. JJM supervised the study and edited the manuscript. All authors read and approved the final manuscript.
